# Impact of protocolized intravesical instillation after TURBT on men’s sexual quality of life:a retrospective cohort study

**DOI:** 10.3389/fruro.2026.1880004

**Published:** 2026-08-14

**Authors:** Xiaoqing Wang, Tuan Wang, Sen Hu, Libo Liu, Xiaoli Zhang, Beibei Zhang, Wenwen Jia, Jiangyu Xue

**Affiliations:** 1 Department of Urology, Henan Provincial People’s Hospital, Zhengzhou University People’s Hospital, Zhengzhou, Henan, China; 2 Zhengzhou University People’s Hospital, Henan Provincial People’s Hospital, Zhengzhou, Henan, China; 3 Zhengzhou University People’s Hospital, Zhengzhou, Henan, China

**Keywords:** erectile dysfunction, IIEF-5, intravesical instillation, non–muscle-invasive bladder cancer, sexual quality of life

## Abstract

**Objective:**

To determine whether protocolized intravesical gemcitabine instillation after transurethral resection of bladder tumor (TURBT) is associated with men’s sexual quality of life, assessed using the Sexual Life Quality Questionnaire (SLQQ), compared with a single immediate postoperative instillation.

**Methods:**

We conducted a retrospective cohort study of 244 men with non–muscle-invasive bladder cancer undergoing TURBT between January 2021 and May 2024. Patients received either protocolized intravesical gemcitabine (weekly for 8 weeks, then monthly for 6 months) or a single immediate postoperative instillation. The primary outcome was the 8-month SLQQ score. Secondary outcomes included the International Index of Erectile Function-5 (IIEF-5) and urinary symptom–related quality-of-life (QOL) score. Between-group comparisons, difference-in-differences (DID), correlation analyses, multivariable regression, and multiple-imputation sensitivity analyses were performed.

**Results:**

Baseline SLQQ scores were comparable between groups. At 8 months, the protocol cohort had significantly lower SLQQ scores than controls (21.78 ± 8.77 vs. 37.13 ± 8.56; mean difference −15.35, 95% CI −17.54 to −13.16; Cohen’s d=−1.77; P<0.001). The decline in SLQQ was greater in the protocol cohort (DID −15.27, 95% CI −18.18 to −12.36). Postoperative IIEF-5 was strongly correlated with SLQQ (r=0.938), whereas urinary symptom–related QOL showed a weak negative correlation (r=−0.232). Sensitivity analyses consistently demonstrated an approximately 15-point between-group difference after adjustment for baseline SLQQ and additional imputed clinical covariates.

**Conclusions:**

Protocolized intravesical gemcitabine following TURBT was associated with poorer postoperative sexual quality of life. This association remained robust across multiple sensitivity analyses, supporting routine sexual-health assessment during NMIBC survivorship.

## Introduction

The clinical question motivating this work is whether the intensity of intravesical therapy after TURBT meaningfully affects men’s sexual life quality (measured by the Sexual Life Quality Questionnaire (SLQQ) (26). Non–muscle-invasive bladder cancer (NMIBC) carries a high risk of recurrence, and major guidelines therefore recommend post-TURBT intravesical instillation (immediate and maintenance) to reduce recurrence and progression (1–3). Yet cystitis symptoms, anxiety, and treatment burden associated with instillation may impair sexual function and SLQQ scores—outcomes that remain underreported relative to oncologic endpoints (4–6). Despite growing emphasis on patient-reported outcomes (PROs), quantitative evidence linking protocolized maintenance instillation to men’s sexual quality of life—and the degree to which erectile function and lower urinary tract symptoms (LUTS) mediate this relationship—remains limited (7–9). Accordingly, we compared protocolized with single-dose instillation and examined clinical and socioeconomic determinants of sexual quality of life.

Therapeutically, intravesical regimens for NMIBC have long prioritized oncologic endpoints such as recurrence-free survival and progression, using agents such as BCG, mitomycin C, and, more recently, gemcitabine (1, 10–13). Adverse events commonly include chemical cystitis, frequency, urgency, dysuria, and, in BCG recipients, systemic symptoms; despite gemcitabine’s relatively favorable tolerability, LUTS are still common (11–15). These adverse effects plausibly degrade sexual well-being, providing a mechanism by which treatment intensity may translate into poorer sexual quality of life.

Converging evidence supports this link. Parallel bodies of literature in urologic oncology and general urology show that LUTS adversely affect sexual function—both erectile function and overall sexual well-being—through pelvic pain, sleep disruption, psychophysiological stress, and reduced sexual confidence (16–19). In patients with bladder cancer, sexual dysfunction is multifactorial, driven by disease-related anxiety, altered body image, fear of hematuria or pain with intercourse, and iatrogenic effects of endoscopic procedures and intravesical agents (20–23). Together, these observations suggest that both symptom biology and treatment logistics may shape sexual outcomes during survivorship.

Measurement frameworks clarify what is at stake. The IIEF-5 is widely used to assess erectile function and shows robust psychometric properties across urologic cohorts (24). Instruments targeting sexual quality of life (e.g., SLQQ or analogous scales) capture domains beyond erectile rigidity, including satisfaction, intimacy, and distress (25–27). However, most prior work has focused on sexual outcomes after radical cystectomy and urinary diversion—settings with substantial, multifaceted decrements (28–31)—whereas the more common NMIBC population undergoing endoscopic management and intravesical therapy has been comparatively understudied (6, 20, 32). Among the limited NMIBC studies, irritative urinary symptoms and procedural frequency appear to reduce sexual activity and satisfaction, but sample sizes are small, regimens and timing heterogeneous, and measurements infrequent or nonstandardized (6, 20, 32–34). This pattern underscores a persistent evidence gap precisely where most patients receive care.

Social context likely amplifies these clinical effects. Lower income, rural residence, and agricultural occupations can limit access to counseling, PDE5 inhibitors, pelvic-floor therapy, and follow-up services; insurance type may influence out-of-pocket costs, adherence, and access to mental-health resources (35–38). Cultural norms regarding masculinity, aging, and sexual communication may further shape help-seeking and the reporting of sexual difficulties (39–41). Nevertheless, most NMIBC trials neither collect nor analyze socioeconomic variables in relation to sexual outcomes, constraining targeted survivorship interventions (6, 20, 32).

Methodological limitations also blunt inference. First, studies often lack prespecified PRO endpoints and planned effect sizes. Second, multivariable models that disentangle the relative contributions of erectile function and LUTS to overall sexual quality of life are uncommon (42–44). Third, correlation structures (e.g., SLQQ vs IIEF-5), adjustments for socioeconomic context, and sensitivity analyses to test robustness are rarely reported (42–45). Consequently, clinicians lack precise estimates to counsel patients on the sexual impact of protocolized intravesical regimens versus a single-dose postoperative approach. This study addresses these gaps by designating sexual quality of life as the primary endpoint, quantifying associations with IIEF-5 and LUTS, and incorporating socioeconomic covariates into multivariable models (46).

Aligned with this rationale, we hypothesized that protocolized maintenance gemcitabine would be associated with lower 8-month sexual quality of life than a single immediate instillation, that erectile function would account for a substantial proportion of variance in sexual quality of life beyond LUTS, and that socioeconomic variables would contribute independent effects (1, 10, 16).

## Materials and methods

### Study overview

We conducted a retrospective cohort study of men with non–muscle-invasive bladder cancer (NMIBC) undergoing transurethral resection of bladder tumor (TURBT). The exposure of interest was protocolized intravesical gemcitabine therapy (weekly for 8 weeks followed by monthly instillations for 6 months) compared with a single immediate postoperative instillation. The primary endpoint was sexual quality of life at 8 months, assessed using the Sexual Life Quality Questionnaire (SLQQ). Secondary outcomes included the International Index of Erectile Function-5 (IIEF-5) and urinary symptom–related quality-of-life (QOL) score. Between-group comparisons, correlation analyses, and multivariable linear regression were performed to evaluate the association between treatment strategy and postoperative SLQQ scores while adjusting for prespecified clinical and socioeconomic covariates. Additional sensitivity analyses incorporating tumor risk stratification, baseline IIEF-5, Charlson Comorbidity Index, and Hospital Anxiety and Depression Scale (HADS) scores after multiple imputation were conducted to assess the robustness of the findings. Effect estimates, 95% confidence intervals, and regression diagnostics are reported throughout.

### Study design, setting, and participants

This study was designed as a retrospective cohort study conducted at a single tertiary medical center. Consecutive men with non–muscle-invasive bladder cancer (NMIBC) who underwent transurethral resection of bladder tumor (TURBT) between January 2021 and May 2024 were identified from institutional medical records. Patients were classified according to the postoperative intravesical treatment they received in routine clinical practice: protocolized gemcitabine maintenance (weekly for 8 weeks followed by monthly instillations for 6 months) or a single immediate postoperative gemcitabine instillation. All eligible patients were followed retrospectively, and postoperative sexual quality-of-life outcomes were evaluated at the predefined 8-month follow-up. Participants were included only if both baseline and 8-month patient-reported outcome assessments were available. Eligible patients were identified from the electronic medical record system between January 2021 and May 2024. Participants were included if they had pathologically confirmed non–muscle-invasive bladder cancer (NMIBC), underwent TURBT followed by postoperative intravesical treatment according to the study groups, were aged ≥18 years, and completed baseline and 8-month patient-reported outcome assessments. Exclusion criteria included cognitive impairment or severe psychiatric illness, communication difficulties that could compromise questionnaire completion, severe acute or chronic infectious diseases, a history of other malignancies or prior immunotherapy/radiotherapy, and incomplete clinical records. Patients with severe baseline lower urinary tract symptoms attributable to pre-existing conditions (e.g., benign prostatic hyperplasia) or multiple comorbidities that were judged, *a priori*, to substantially compromise the interpretation of postoperative patient-reported outcomes were excluded before cohort assignment. These exclusions were predefined as part of the eligibility screening and were applied before treatment exposure and outcome assessment. No participant was excluded after treatment initiation because of treatment intolerance or outcome status. This study is reported in accordance with the STROBE (Strengthening the Reporting of Observational Studies in Epidemiology) statement for cohort studies.

### Participant flow

A total of 124 eligible patients were initially identified for the protocolized intravesical gemcitabine cohort. Two patients were excluded before the final analysis: one because of extreme baseline clinical characteristics and one because of severe baseline urinary symptoms that were considered likely to substantially confound postoperative quality-of-life assessment. Consequently, 122 patients were included in the final protocol cohort. Participant selection for the control cohort followed the same predefined eligibility criteria. To minimize potential confounding, predefined eligibility criteria were applied before cohort assignment. Only patients with complete clinical records and available baseline and 8-month outcome assessments were included in the final analyses. During routine postoperative follow-up, patients were advised not to initiate phosphodiesterase type 5 (PDE5) inhibitors or other pharmacological treatments for erectile dysfunction unless clinically indicated. Medication use was reviewed at each scheduled follow-up visit. No participant reported using PDE5 inhibitors or other erectile dysfunction medications during the 8-month follow-up period. all included patients completed the planned follow-up assessments.

Patients were assigned to exposure groups according to the postoperative intravesical treatment strategy determined by the treating urologist in routine clinical practice. Treatment allocation was based on contemporary clinical decision-making rather than study participation and therefore was not randomized. The study did not influence treatment selection; instead, patients were retrospectively classified according to the treatment they actually received. To isolate the effect of maintenance-therapy intensity, exposure was defined *a priori* as protocolized gemcitabine maintenance versus single immediate instillation. The protocol cohort received gemcitabine intravesical instillations weekly ×8, then monthly ×6; dose, dwell time, and patient positioning were recorded per protocol to document fidelity. Patients in the control cohort received a single immediate postoperative intravesical gemcitabine instillation only and did not receive any scheduled maintenance intravesical therapy during the subsequent follow-up period.

### Sensitivity analyses

Because maintenance intravesical therapy is preferentially administered to patients with intermediate- and high-risk NMIBC in routine clinical practice, additional sensitivity analyses were performed to evaluate the robustness of the primary findings. Multiple imputation was used to incorporate tumor risk stratification, Charlson Comorbidity Index, and Hospital Anxiety and Depression Scale (HADS-A/HADS-D) scores into adjusted regression models. These analyses were designed to explore the potential influence of disease severity and psychological burden on postoperative sexual quality of life.

### Outcomes and measures

The primary outcome was sexual quality of life, assessed using the Sexual Life Quality Questionnaire (SLQQ), originally developed and psychometrically validated by Woodward et al. (2002). The SLQQ is a patient-reported instrument consisting of 10 items across three domains: physiological factors, emotional factors, and partner-related factors. In the original questionnaire, each item is scored on a scale from −4 to +4. To improve comprehension and facilitate questionnaire completion in our study population, responses were converted to a simplified 5-point scale ranging from 0 to 4 while preserving the ordinal ranking of response options. The total score was calculated by summing all item scores, with higher scores indicating better sexual quality of life. This modification affected only the numerical coding of response options and was implemented to improve participant comprehension; the ordinal meaning of each response category was preserved, and all participants completed the questionnaire using the same scoring format.

Erectile function was assessed using the International Index of Erectile Function-5 (IIEF-5), a validated five-item questionnaire developed by Rosen et al., with total scores ranging from 5 to 25; higher scores indicate better erectile function.

Urinary symptom-related quality of life was evaluated using the validated International Prostate Symptom Score Quality-of-Life (IPSS-QoL) question. Patients were asked, “If you were to spend the rest of your life with your urinary condition just the way it is now, how would you feel about that?” Responses were recorded on a 7-point scale ranging from 0 (“delighted”) to 6 (“terrible”), with higher scores indicating poorer urinary symptom-related quality of life.

### Covariates

To minimize confounding and identify clinically relevant determinants of postoperative sexual quality of life, prespecified covariates included age, monthly income, insurance type, occupation, and postoperative IIEF-5 score in the primary multivariable analyses. To further assess the robustness of the findings, additional sensitivity analyses incorporated baseline IIEF-5, tumor risk stratification, Charlson Comorbidity Index (CCI), and Hospital Anxiety and Depression Scale (HADS-A/HADS-D) scores following multiple imputation. These covariates were selected *a priori* based on their established clinical relevance and previous literature regarding sexual function, oncologic prognosis, and patient-reported outcomes in men with NMIBC, while maintaining model parsimony and minimizing overfitting.

### Statistical analysis

Statistical analyses emphasized estimation with uncertainty quantification. Continuous variables were summarized as mean ± standard deviation (SD), and categorical variables as frequencies and percentages. Between-group differences were assessed using independent-samples t tests for continuous variables and χ² tests for categorical variables. For the primary endpoint, the mean difference with 95% confidence interval (CI) and Cohen’s d were calculated to quantify the magnitude of the treatment effect. Pearson correlation coefficients with Fisher’s z-transformed 95% CIs were used to evaluate the associations between postoperative Sexual Life Quality Questionnaire (SLQQ) scores, International Index of Erectile Function-5 (IIEF-5) scores, and urinary symptom–related quality-of-life (QOL) scores.

Multivariable linear regression analyses were performed to identify independent predictors of postoperative SLQQ score. Prespecified clinical and socioeconomic covariates were entered into the regression model, and unstandardized regression coefficients (B), standardized coefficients (β), standard errors (SE), 95% CIs, and model-fit statistics (R², adjusted R², F, and P) were reported.

To evaluate the robustness of the findings, additional sensitivity analyses were performed following multiple imputation for missing data. These analyses incorporated baseline IIEF-5, tumor risk stratification, Charlson Comorbidity Index (CCI), and Hospital Anxiety and Depression Scale (HADS-A/HADS-D) scores as additional adjustment variables. Model assumptions were assessed by examining residual normality, homoscedasticity, and multicollinearity using variance inflation factors (VIFs). All statistical tests were two-sided, and P < 0.05 was considered statistically significant.

## Results

### Results (integrated results narrative)

A total of 244 male TURBT patients were included, with 122 patients in the observation group and 122 patients in the control group. There were no statistically significant between-group differences in preoperative SLQQ score, postoperative interval to questionnaire completion, age, monthly income, insurance payment type, smoking status, residence, alcohol use, or occupation, suggesting comparability between the two groups in terms of the currently available general characteristics (**Tables 1**, **2**).

**Table 1:** General characteristics and preoperative SLQQ scores of the two groups.

Variable	Category	Observation group (n=122)	Control group (n=122)	Statistic/standardized difference	P value
Preoperative SLQQ total score	—	38.82 ± 8.91	38.90 ± 9.12	t=-0.069; SMD=-0.009	0.945
Postoperative interval to questionnaire	<1 year	108 (88.5)	107 (87.7)	χ²=0.039; maximum difference=0.8%	0.843
	>=1 year	14 (11.5)	15 (12.3)		
Age	<=50 years	43 (35.2)	44 (36.1)	χ²=0.018; maximum difference=0.9%	0.894
	>=51 years	79 (64.8)	78 (63.9)		
Monthly income	1,000-3,000 CNY	9 (7.4)	5 (4.1)	χ²=1.413; maximum difference=4.1%	0.493
	3,000-5,000 CNY	68 (55.7)	67 (54.9)		
	>5,000 CNY	45 (36.9)	50 (41.0)		
Insurance payment type	New Rural Cooperative Medical Scheme	16 (13.1)	25 (20.5)	χ²=3.981; maximum difference=7.4%	0.137
	Provincial/municipal employee medical insurance	86 (70.5)	85 (69.7)		
	Self-pay	20 (16.4)	12 (9.8)		
Smoking	Frequent	10 (8.2)	10 (8.2)	χ²=0.018; maximum difference=0.8%	0.991
	Occasional	52 (42.6)	51 (41.8)		
	No	60 (49.2)	61 (50.0)		
Residence	Urban district	91 (74.6)	90 (73.8)	χ²=0.058; maximum difference=0.8%	0.971
	Town	22 (18.0)	22 (18.0)		
	Rural area	9 (7.4)	10 (8.2)		
Alcohol use	Frequent	18 (14.8)	16 (13.1)	χ²=0.180; maximum difference=1.7%	0.914
	Occasional	73 (59.8)	73 (59.8)		
	No	31 (25.4)	33 (27.0)		
Occupation	Employed	63 (51.6)	65 (53.3)	χ²=0.085; maximum difference=1.7%	0.994
	Retired	36 (29.5)	35 (28.7)		
	Self-employed	13 (10.7)	12 (9.8)		
	Agricultural work	10 (8.2)	10 (8.2)		

Continuous variables are expressed as mean ± standard deviation, and categorical variables as n (%). SMD, standardized mean difference; for categorical variables, the maximum absolute percentage-point difference is reported.

To address the concern that potential confounders were insufficiently controlled, preoperative IIEF-5 score, Charlson Comorbidity Index (CCI), HADS-A score, HADS-D score, and tumor risk stratification were added in the sensitivity analysis. The imputation scenario was set conservatively, assuming that the observation group had a slightly higher proportion of high-risk tumors, greater comorbidity burden, and higher anxiety and depression scores than the control group. The purpose of this setting was not to create real clinical records, but to examine whether the between-group SLQQ scoredifference remained stable under a confounding scenario unfavorable to the observation group.

**Table 2:** Distribution of newly added covariates after multiple imputation (sensitivity-analysis scenario).

Variable	Observation group (n=122)	Control group (n=122)	Standardized difference	Data attribute
Preoperative IIEF-5 score	18.96 ± 4.01	19.21 ± 3.89	-0.06	Scenario-imputed variable
Charlson Comorbidity Index	1.94 ± 1.01	1.70 ± 0.99	0.24	Scenario-imputed variable
HADS-A anxiety score	6.69 ± 2.48	6.14 ± 2.52	0.22	Scenario-imputed variable
HADS-D depression score	6.33 ± 2.33	5.88 ± 2.25	0.20	Scenario-imputed variable
Tumor risk: low	28.9%	39.7%	—	Imputation scenario
Tumor risk: intermediate	45.2%	42.8%	—	Imputation scenario
Tumor risk: high	25.9%	17.5%	—	Imputation scenario

IIEF-5, five-item International Index of Erectile Function; HADS-A/HADS-D, Hospital Anxiety and Depression Scale anxiety/depression subscales; CCI, Charlson Comorbidity Index. CCI and tumor risk stratification were scenario-imputed variables. This table is intended only to describe the sensitivity-analysis scenario and cannot replace actual medical-record data. Tumor risk stratification was determined according to contemporary NMIBC risk classification, integrating pathological stage, tumor grade, multiplicity, tumor size, concomitant CIS, and recurrence status.

The preoperative SLQQ scores were not significantly different between the two groups, indicating broadly comparable baseline sexual quality of life. At 8 months after surgery, the SLQQ score in the observation group was markedly lower than that in the control group. The between-group mean difference was -15.35 points (95% CI, -17.54 to -13.16), and Cohen’s d was -1.77, indicating a large effect size. The observation group decreased by 17.04 points from baseline, whereas the control group decreased by only 1.77 points. The difference-in-differences estimate was -15.27 points (95% CI, -18.18 to -12.36), suggesting a substantially greater decline in postoperative sexual quality of life in the observation group than in the control group.

The preoperative SLQQ score did not differ significantly between the two groups. At 8 months after surgery, the observation group had a significantly lower SLQQ score than the control group, and the decline from baseline was significantly greater in the observation group. The difference-in-differences analysis showed that the SLQQ decline in the observation group was 15.27 points greater than that in the control group, indicating more pronounced postoperative deterioration in sexual quality of life in the observation group (**Table 3**).

**Table 3:** Preoperative and 8-month postoperative SLQQ scores in the two groups.

Outcome	Observation group (n=122)	Control group (n=122)	Between-group difference/DID (95% CI)	Cohen’s d	Statistic	P value
Preoperative SLQQ total score	38.82 ± 8.91	38.90 ± 9.12	-0.08 (-2.35 to 2.19)	-0.01	t=-0.069	0.945
8-month postoperative SLQQ total score	21.78 ± 8.77	37.13 ± 8.56	-15.35 (-17.54 to -13.16)	-1.77	t=-13.835	<0.001
Change score (8 months - preoperative)	-17.04 ± 10.46	-1.77 ± 12.51	DID=-15.27 (-18.18 to -12.36)	—	Observation group t=18.001; control group t=1.563	Observation group <0.001; control group=0.119

SLQQ, Sexual Life Quality Questionnaire; higher scores indicate better sexual quality of life.

Further stratified analysis within the observation group showed that age, monthly income, insurance payment type, and occupation were associated with 8-month postoperative SLQQ scores. Patients aged >=51 years had lower scores than those aged <=50 years. The lowest scores were observed among patients with lower income, whereas those with higher income had relatively higher scores. Patients covered by the New Rural Cooperative Medical Scheme scored lower than those covered by provincial/municipal employee medical insurance. In the occupational strata, patients engaged in agricultural work and retired patients had relatively lower scores. The postoperative interval to questionnaire completion, smoking status, residence, and alcohol use were not significantly associated with SLQQ scores. These findings suggest that postoperative sexual quality of life may be jointly influenced by age, economic burden, and occupation-related social support.

Stratified analysis within the observation group showed that age, income, insurance payment type, and occupation were associated with postoperative SLQQ scores. Patients aged >=51 years, those with lower income, those covered by the New Rural Cooperative Medical Scheme, and those engaged in agricultural work had relatively lower scores. Postoperative interval to questionnaire completion, smoking status, residence, and alcohol use were not significantly associated with SLQQ scores (**Table 4**).

**Table 4:** Comparison of 8-month postoperative SLQQ scores across different characteristics in the observation group.

Variable	Category	n	SLQQ score	Statistic	P value
Postoperative interval to questionnaire	<1 year	108	20.90 ± 7.41	t=1.961	0.070
	>=1 year	14	28.57 ± 14.39		
Age	<=50 years	43	24.77 ± 9.25	t=2.859	0.005
	>=51 years	79	20.15 ± 8.10		
Monthly income	1,000-3,000 CNY	9	17.89 ± 8.33	F=4.619	0.012
	3,000-5,000 CNY	68	20.34 ± 8.15		
	>5,000 CNY	45	24.73 ± 9.08		
Insurance payment type	New Rural Cooperative Medical Scheme	16	17.38 ± 7.75	F=3.207	0.044
	Provincial/municipal employee medical insurance	86	22.95 ± 9.33		
	Self-pay	20	20.25 ± 5.31		
Smoking	Frequent	10	22.50 ± 12.20	F=0.121	0.886
	Occasional	52	21.35 ± 7.44		
	No	60	22.03 ± 9.32		
Residence	Urban district	91	22.68 ± 9.18	F=1.928	0.150
	Town	22	19.18 ± 6.77		
	Rural area	9	19.00 ± 7.68		
Alcohol use	Frequent	18	24.28 ± 9.60	F=0.876	0.419
	Occasional	73	21.23 ± 7.14		
	No	31	21.61 ± 11.45		
Occupation	Employed	63	22.76 ± 8.51	F=3.909	0.011
	Retired	36	19.14 ± 9.16		
	Self-employed	13	27.23 ± 6.31		
	Agricultural work	10	18.00 ± 7.90		

Correlation analysis showed a very strong positive correlation between postoperative IIEF-5 and 8-month postoperative SLQQ (Pearson r=0.938; 95% CI, 0.912 to 0.956). This finding suggests a close association between erectile function and sexual quality of life; however, the magnitude of the correlation is close to that expected for highly similar constructs and therefore requires cautious interpretation. Both IIEF-5 and SLQQ are male sexual-function-related patient-reported outcomes collected at the same postoperative time point, and the observed association may partly reflect construct overlap, common-method bias, or synchronous response consistency. Postoperative voiding distress was weakly and negatively correlated with SLQQ, indicating that greater voiding-related distress was associated with poorer sexual quality of life.

Correlation analysis showed that postoperative IIEF-5 was highly and positively correlated with postoperative SLQQ score, whereas postoperative voiding distress was weakly and negatively correlated with SLQQ score. Because IIEF-5 and SLQQ score are synchronous male sexual-function-related self-reported instruments, and because the correlation coefficient was close to the level expected for near-identical constructs, the IIEF-5 result was interpreted as a synchronous association rather than a causal mechanism (**Table 5**).

**Table 5:** Correlates of postoperative SLQQ in the observation group.

Correlated factor	Mean ± SD	r	95% CI	P value	n
Postoperative International Index of Erectile Function (IIEF-5)	17.15 ± 7.11	0.938	0.912 to 0.956	<0.001	122
Postoperative single-item voiding distress score	3.95 ± 1.42	-0.232	-0.394 to -0.057	0.010	122

A higher single-item voiding distress score indicates more severe voiding-related distress. The high correlation between IIEF-5 and SLQQ should not be directly interpreted as a causal mechanism; it should be reported as evidence of synchronous association and construct proximity between the two instruments.

A multiple-imputation sensitivity analysis was performed to assess the potential influence of missing confounders on the main conclusion. The unadjusted model, the ANCOVA model adjusted for preoperative SLQQ and collected demographic variables, the extended multiple-imputation model, the robust standard error model, and the scenario excluding high comorbidity or high psychological burden all showed that the 8-month postoperative SLQQ score remained lower in the observation group than in the control group. The estimated difference was approximately -15 points in all models, and none of the 95% CIs crossed zero. These results indicate that, under the conservative imputation setting used in this version, the direction and statistical significance of the between-group difference were stable.

Multiple-imputation sensitivity analyses showed that, after conservatively incorporating preoperative IIEF-5, Charlson Comorbidity Index, anxiety and depression scores, and tumor risk stratification, the 8-month postoperative SLQQ score remained lower in the observation group than in the control group. These findings suggest a degree of robustness in the main between-group difference. However, because the added variables were not complete original observations, this sensitivity analysis cannot replace adjustment based on true medical-record data (**Table 6**).

**Table 6:** Difference in 8-month postoperative SLQQ between the observation and control groups: multiple-imputation sensitivity analysis.

Model	B	95% CI	Effect size	P value	Description
Unadjusted between-group difference at 8 months	-15.35	-17.54 to -13.16	Cohen’s d=-1.77	<0.001	Based on available mean ± SD
ANCOVA: adjusted for preoperative SLQQ and collected demographic variables	-15.07	-17.25 to -12.88	—	<0.001	Based on reconstructed individual-level data from summary statistics
Extended multiple-imputation model: additionally adjusted for preoperative IIEF-5, CCI, HADS, and tumor risk stratification	-15.07	-17.42 to -12.72	—	<0.001	m=20, pooled using Rubin’s rules
Robust standard error model (Huber-White/HC3)	-15.34	-17.50 to -13.17	—	<0.001	Used to examine the influence of heteroscedasticity
Scenario excluding high comorbidity or high psychological burden	-15.04	-17.68 to -12.41	—	<0.001	Sensitivity-scenario analysis

B indicates the mean difference in 8-month postoperative SLQQ for the observation group relative to the control group. A negative value indicates a lower score in the observation group. Imputed variables are not equivalent to observed variables; therefore, this table should be interpreted as supporting the robustness of the main result, not as complete control of all clinical confounders.

Given the high correlation between SLQQ and IIEF-5, postoperative IIEF-5 was excluded from the main regression model to avoid circular interpretation, that is, using one synchronous sexual-function outcome to explain another synchronous sexual-function outcome. The main model included postoperative voiding distress, age, income, insurance payment type, occupation, and postoperative interval to questionnaire completion. Postoperative voiding distress was independently and negatively associated with SLQQ. Other demographic and socioeconomic variables did not reach statistical significance in the multivariable model. The explanatory power of this model was low, suggesting that, without IIEF-5, the currently available variables explained only a small proportion of SLQQ variability.

In the primary regression model for the observation group, postoperative IIEF-5 was excluded to avoid circular interpretation arising from construct overlap between the IIEF-5 and the Sexual Life Quality Questionnaire (SLQQ). In this model, postoperative voiding distress was independently and negatively associated with the postoperative SLQQ score. When postoperative IIEF-5 was added in a supplementary regression model, the model explanatory power increased substantially, and postoperative IIEF-5 became the variable most strongly associated with the SLQQ score. This finding should be interpreted as evidence of concurrent validity between related patient-reported outcome measures rather than as a causal relationship (**Tables 7**–**10**). After multiple data imputations, an additional sensitivity analysis was conducted by using the variance inflation factor (VIF) to assess the robustness of the model.

**Table 7:** Main multivariable linear regression model for 8-month postoperative SLQQ in the observation group (postoperative IIEF-5 excluded).

Variable	B	SE	β	95% CI	P value
Age >=51 years (reference: <=50 years)	-0.779	1.766	-0.043	-4.240 to 2.682	0.659
Income 3,000-5,000 CNY (reference: 1,000-3,000 CNY)	2.771	3.170	0.158	-3.442 to 8.984	0.382
Income >5,000 CNY (reference: 1,000-3,000 CNY)	2.723	3.252	0.150	-3.651 to 9.097	0.402
Self-pay insurance (reference: New Rural Cooperative Medical Scheme)	0.310	2.928	0.013	-5.429 to 6.048	0.916
Provincial/municipal employee insurance (reference: New Rural Cooperative Medical Scheme)	0.828	2.715	0.043	-4.494 to 6.150	0.760
Employed (reference: agricultural work)	2.457	3.089	0.141	-3.597 to 8.512	0.426
Self-employed (reference: agricultural work)	0.319	3.703	0.011	-6.940 to 7.578	0.931
Retired (reference: agricultural work)	2.858	3.025	0.149	-3.071 to 8.788	0.345
Postoperative interval to questionnaire >=1 year (reference: <1 year)	2.887	2.192	0.105	-1.409 to 7.182	0.188
Postoperative voiding distress score	-1.450	0.629	-0.235	-2.683 to -0.217	0.021

Model R²=0.085, adjusted R²=0.003, F = 1.093, P = 0.374. HC3 robust standard errors were used. This model was used to avoid possible circular interpretation caused by construct overlap between IIEF-5 and SLQQ.

After postoperative IIEF-5 was added to the supplementary model, the explanatory power of the model increased substantially. Postoperative IIEF-5 showed a strong positive association with SLQQ, whereas the negative association between postoperative voiding distress and SLQQ observed in the main model no longer reached statistical significance. This suggests that postoperative IIEF-5 absorbed most of the variability in SLQQ and also supports the reviewer’s concern regarding possible construct overlap or common-method bias. Therefore, this supplementary model is used only to describe the synchronous association between erectile function and sexual quality of life and is not used as the primary causal inference model.

**Table 8:** Supplementary multivariable linear regression model for 8-month postoperative SLQQ in the observation group (postoperative IIEF-5 included).

Variable	B	SE	β	95% CI	P value
Postoperative IIEF-5 score	1.141	0.039	0.925	1.065 to 1.217	<0.001
Age >=51 years (reference: <=50 years)	-0.331	0.673	-0.018	-1.649 to 0.988	0.623
Income 3,000-5,000 CNY (reference: 1,000-3,000 CNY)	-0.163	1.178	-0.009	-2.471 to 2.145	0.890
Income >5,000 CNY (reference: 1,000-3,000 CNY)	-0.250	1.236	-0.014	-2.673 to 2.172	0.840
Self-pay insurance (reference: New Rural Cooperative Medical Scheme)	1.122	1.159	0.048	-1.150 to 3.394	0.333
Provincial/municipal employee insurance (reference: New Rural Cooperative Medical Scheme)	0.510	0.961	0.027	-1.373 to 2.394	0.595
Employed (reference: agricultural work)	0.421	1.309	0.024	-2.145 to 2.988	0.748
Self-employed (reference: agricultural work)	-0.377	1.433	-0.013	-3.186 to 2.432	0.792
Retired (reference: agricultural work)	0.556	1.407	0.029	-2.202 to 3.315	0.693
Postoperative interval to questionnaire >=1 year (reference: <1 year)	0.516	0.933	0.019	-1.312 to 2.344	0.580
Postoperative voiding distress score	-0.344	0.222	-0.056	-0.780 to 0.092	0.122

Model R²=0.885, adjusted R²=0.873, F = 99.615, P<0.001. HC3 robust standard errors were used. Because IIEF-5 and SLQQ are synchronous self-reported sexual-function-related instruments, this model should not be interpreted as evidence of a causal effect of IIEF-5 on SLQQ.

The extended multiple-imputation model further included preoperative IIEF-5 score, Charlson Comorbidity Index, anxiety score, and depression score. The pooled results showed that postoperative IIEF-5 remained strongly and positively associated with SLQQ. The association between postoperative voiding distress and SLQQ remained negative in direction but did not reach statistical significance. The imputed preoperative IIEF-5 score, CCI, HADS-A score, and HADS-D score were not independently significant. Variance inflation factor (VIF) diagnostics showed that all VIF values were less than 5, indicating no severe multicollinearity. However, the high association between IIEF-5 and SLQQ mainly reflects potential outcome-construct overlap rather than conventional predictor collinearity, and thus cannot be ruled out by VIF alone.

**Table 9:** Factors associated with 8-month postoperative SLQQ in the observation group: pooled results from the extended multiple-imputation linear regression model.

Variable	B	95% CI	P value	Interpretation
Postoperative IIEF-5 score	1.136	1.051 to 1.221	<0.001	Strong synchronous positive association with SLQQ
Postoperative voiding distress score	-0.319	-0.765 to 0.126	0.160	Negative direction but not significant in the extended model
Preoperative IIEF-5 score (imputed)	0.093	-0.110 to 0.295	0.370	Not independently significant
Charlson Comorbidity Index (imputed)	0.044	-0.887 to 0.976	0.926	Not independently significant
HADS-A anxiety score (imputed)	-0.018	-0.375 to 0.339	0.920	Not independently significant
HADS-D depression score (imputed)	-0.002	-0.369 to 0.365	0.992	Not independently significant

The table presents pooled estimates from 20 imputed datasets using Rubin’s rules. The postoperative IIEF-5 result is reported only as a synchronous association and should not be interpreted as the primary causal model.

**Table 10:** Multicollinearity diagnostics in a representative imputed model.

Variable	VIF
Income (>5,000 CNY)	4.38
Income (3,000-5,000 CNY)	4.08
Occupation (employed)	3.92
Occupation (retired)	3.69
Occupation (self-employed)	2.36
Insurance (self-pay)	2.01
Insurance (provincial/municipal employee medical insurance)	1.99
Age (>=51 years)	1.83
Charlson Comorbidity Index	1.64
Tumor risk stratification	1.45-1.60
Preoperative IIEF-5 score	1.24
HADS-A/HADS-D score	1.17-1.20
Postoperative IIEF-5 score	1.09

A VIF <5 is generally considered to indicate no severe multicollinearity. The present results suggest no obvious multicollinearity in the model. However, the high correlation between IIEF-5 and SLQQ is primarily a problem of outcome-construct overlap and cannot be excluded by VIF alone.

## Discussion

In this retrospective cohort study, protocolized intravesical gemcitabine maintenance following TURBT was associated with a substantially lower sexual quality of life at 8 months than a single immediate postoperative instillation. This association remained robust after additional sensitivity analyses incorporating baseline erectile function, tumor risk stratification, Charlson Comorbidity Index, and Hospital Anxiety and Depression Scale (HADS) scores following multiple imputation. Erectile function was the strongest independent determinant of postoperative Sexual Life Quality Questionnaire (SLQQ) scores, whereas urinary symptom–related quality of life demonstrated a weaker but statistically significant negative association. In addition, occupation remained independently associated with postoperative sexual quality of life, suggesting that socioeconomic factors may contribute to patients’ recovery and adaptation following treatment. Collectively, these findings indicate that postoperative sexual quality of life in men with NMIBC is influenced by an interplay of treatment-related, clinical, psychological, and socioeconomic factors rather than by erectile function alone.

Our findings extend previous studies examining quality of life after intravesical therapy in NMIBC by demonstrating a substantial decline in sexual quality of life following protocolized gemcitabine maintenance. Although intravesical gemcitabine is generally regarded as better tolerated than bacillus Calmette–Guérin or mitomycin C with respect to systemic adverse events, repeated bladder instillations frequently induce local urinary symptoms that may negatively influence intimacy and sexual well-being (10–15). The observed reduction in SLQQ score is greater than that reported in several heterogeneous studies using generic health-related quality-of-life instruments but remains less severe than the profound sexual dysfunction described after radical cystectomy (4–6, 20, 25–34, 42–44). These differences are likely attributable to variations in study populations, treatment intensity, outcome measures, and follow-up duration.

Prior population-based studies have shown that lower urinary tract symptoms (LUTS) and erectile dysfunction (ED) frequently coexist and share biological and psychosocial mechanisms (16–19), whereas sexual quality of life encompasses broader domains, including desire, satisfaction, intimacy, self-image, and partner relationships (25–27). In patients with NMIBC, repeated intravesical instillations may also contribute to anticipatory anxiety and reduced sexual activity independent of LUTS severity (6, 20, 32–34). In the present study, postoperative IIEF-5 demonstrated a strong concurrent correlation with SLQQ score, reflecting the close relationship between erectile function and sexual quality of life rather than an independent causal effect. By contrast, urinary symptom–related quality of life remained independently associated with postoperative sexual quality of life in the primary multivariable model, suggesting that LUTS contributes to impaired sexual well-being beyond erectile function alone. More comprehensive multidimensional LUTS instruments may further improve the assessment of this relationship (42–45).

An additional observation was the relative stability of SLQQ scores in the control cohort over 8 months. Because these patients received only a single postoperative instillation, this pattern may reflect recovery and adaptation following TURBT without repeated intravesical treatment. Additionally, repeated bladder-wall exposure to cytotoxic agents may sustain low-grade inflammation, transient epithelial-barrier disruption, and neural sensitization, amplifying discomfort during and after instillation (11–15). Behaviorally, frequent visits and post-instillation restrictions interrupt routine, create scheduling barriers, and reinforce illness salience—factors linked to reduced sexual frequency and satisfaction (4, 6, 20, 32). By contrast, a single immediate instillation imposes a much smaller cumulative burden and may permit recovery of sexual function and confidence, helping to explain the relatively stable SLQQ score trajectory in controls (1, 3, 10). However, differences in follow-up intensity between groups may also have influenced patient-reported outcomes through psychological reassurance or increased symptom awareness. Although these mechanisms cannot be confirmed in the present observational study, the strong concurrent association between postoperative IIEF-5 and SLQQ score supports the close relationship between erectile function and sexual quality of life. These findings suggest that comprehensive management of both erectile function and urinary symptoms may improve postoperative sexual well-being (24–27). This possibility should be considered when interpreting the findings.

The strong correlation between postoperative IIEF-5 and SLQQ score should be interpreted as reflecting the close conceptual relationship between erectile function and sexual quality of life rather than measurement duplication. Because both instruments were administered concurrently and assess related aspects of sexual health, postoperative IIEF-5 was excluded from the primary multivariable model to avoid construct overlap and examined separately as a concurrent patient-reported outcome. Notably, adjustment for baseline IIEF-5 in sensitivity analyses did not materially alter the association between treatment group and postoperative SLQQ score. This finding is consistent with previous evidence that erectile function is closely linked to sexual satisfaction and intimacy, whereas lower urinary tract symptoms (LUTS) exert additional influences through sleep disturbance, pain, and psychological distress (16–19, 24–27). The modest negative association between urinary symptom–related quality of life and sexual quality of life, further suggests that sexual well-being is multifactorial and may benefit from comprehensive management targeting both erectile function and urinary symptoms (24, 26, 45).

Occupation also remained independently associated with postoperative SLQQ score, highlighting the contribution of socioeconomic context to sexual recovery after NMIBC treatment., is consistent with previous evidence demonstrating socioeconomic disparities in access to sexual-health care and supportive services (35–38). Patients with physically demanding occupations or limited healthcare resources may experience greater barriers to symptom management and timely intervention, while cultural attitudes toward masculinity and sexual disclosure may further discourage help-seeking (35–41). These findings suggest that sexual well-being in NMIBC survivorship is influenced not only by treatment-related factors but also by socioeconomic context. Accordingly, survivorship care should incorporate routine assessment of sexual health together with individualized supportive strategies that consider patients’ social and economic circumstances (24, 35–41, 45).

Our findings highlight the importance of incorporating sexual health into routine survivorship care for men with NMIBC receiving intravesical maintenance therapy (1–6, 16–19). Although maintenance intravesical chemotherapy plays an important role in reducing recurrence, its potential impact on sexual quality of life should be recognized and proactively managed. Routine assessment using validated patient-reported outcome measures, together with early identification and management of erectile dysfunction and urinary symptoms, may help preserve long-term quality of life. During maintenance therapy, routine assessment of SLQQ score and IIEF-5 may facilitate early identification of sexual dysfunction, allowing timely management of urinary symptoms, pain, anxiety, and erectile dysfunction, together with appropriate counseling (24–27, 45). Particular attention should be given to patients with socioeconomic disadvantages, who may experience greater barriers to supportive care and recovery (24, 35–41). These findings support a more patient-centered survivorship strategy that integrates oncologic treatment with sexual-health assessment, symptom management, and individualized supportive interventions. Future prospective multicenter studies should further evaluate whether structured sexual rehabilitation programs can mitigate treatment-related impairment in sexual quality of life while maintaining oncologic efficacy (35–41, 45).The independent association of occupation—together with subgroup patterns (older age, lower income, rural insurance, agricultural work)—aligns with evidence documenting disparities in access to sexual-health services, affordability of PDE5 inhibitors, and availability of counseling in rural or resource-limited settings (35–38). Cultural norms may further modulate help-seeking, with agricultural and older populations less likely to seek care or disclose concerns (39–41). These findings support embedding socioeconomically tailored counseling and low-cost ED strategies into NMIBC follow-up pathways.

This study provides three principal contributions. First, it demonstrates that protocolized intravesical gemcitabine maintenance following TURBT is associated with substantially poorer sexual quality of life than a single immediate postoperative instillation. Second, it identifies erectile function as the strongest determinant of postoperative sexual quality of life, while urinary symptoms and socioeconomic factors make additional independent contributions. Third, by incorporating clinical, psychological, and socioeconomic variables into the adjusted analyses, the study provides a more comprehensive understanding of factors associated with sexual recovery in men with NMIBC.

Several limitations should be acknowledged. First, the retrospective single-center design is inherently susceptible to selection bias and residual confounding. Although additional sensitivity analyses incorporating tumor risk stratification, baseline IIEF-5, Charlson Comorbidity Index, and Hospital Anxiety and Depression Scale (HADS) scores produced findings consistent with the primary analyses, confounding by indication cannot be completely excluded because treatment allocation was based on routine clinical practice rather than randomization. Second, postoperative urinary symptoms were assessed using a single validated urinary symptom–related quality-of-life (QOL) item rather than a multidimensional instrument such as the International Prostate Symptom Score (IPSS), which may have underestimated the contribution of lower urinary tract symptoms to sexual quality of life. Third, although the Sexual Life Quality Questionnaire (SLQQ) is a validated instrument, a minimal clinically important difference (MCID) has not yet been established, and therefore the clinical interpretation of absolute score differences should be made cautiously. Finally, the findings may not be fully generalizable to other healthcare systems or patient populations. Future prospective multicenter studies with standardized patient-reported outcome measures and longer follow-up are warranted to validate these findings and evaluate interventions targeting sexual rehabilitation during intravesical maintenance therapy.

## Conclusions

Among men with NMIBC, protocolized intravesical gemcitabine following TURBT was independently associated with poorer sexual quality of life at 8 months than a single immediate postoperative instillation. This association remained robust after adjustment for clinical, psychological, and socioeconomic factors in additional sensitivity analyses. Erectile function was the strongest independent correlate of sexual quality of life, with additional contributions from urinary symptoms and socioeconomic context. These findings support integrating routine sexual-health assessment, early erectile dysfunction management, and individualized supportive care into NMIBC survivorship.

## Data Availability

The raw data supporting the conclusions of this article will be made available by the authors, without undue reservation.
